# Protein disulfide isomerase family member 4 promotes triple-negative breast cancer tumorigenesis and radiotherapy resistance through JNK pathway

**DOI:** 10.1186/s13058-023-01758-6

**Published:** 2024-01-02

**Authors:** Jinqiu Tao, Cailin Xue, Meng Cao, Jiahui Ye, Yulu Sun, Hao Chen, Yinan Guan, Wenjie Zhang, Weijie Zhang, Yongzhong Yao

**Affiliations:** 1 Division of Breast Surgery, Department of General Surgery, Nanjing Drum Tower Hospital, The Affiliated Hospital of Medical School, Nanjing University, Nanjing, 210008 China; 2 Department of Hepatobiliary Surgery, The First Affiliated Hospital of Anhui Medical University, Hefei, 230022 China; 3 Division of Hepatobilliary Surgery, Department of General Surgery, Nanjing Drum Tower Hospital, The Affiliated Hospital of Medical School, Nanjing University, Nanjing, 210008 China

**Keywords:** PDIA4, TNBC, Apoptosis, Radiation resistance, TAXBP1 JNK

## Abstract

**Background:**

Despite radiotherapy ability to significantly improve treatment outcomes and survival in triple-negative breast cancer (TNBC) patients, acquired resistance to radiotherapy poses a serious clinical challenge. Protein disulfide isomerase exists in endoplasmic reticulum and plays an important role in promoting protein folding and post-translational modification. However, little is known about the role of protein disulfide isomerase family member 4 (PDIA4) in TNBC, especially in the context of radiotherapy resistance.

**Methods:**

We detected the presence of PDIA4 in TNBC tissues and paracancerous tissues, then examined the proliferation and apoptosis of TNBC cells with/without radiotherapy. As part of the validation process, xenograft tumor mouse model was used. Mass spectrometry and western blot analysis were used to identify PDIA4-mediated molecular signaling pathway.

**Results:**

Based on paired clinical specimens of TNBC patients, we found that PDIA4 expression was significantly higher in tumor tissues compared to adjacent normal tissues. In vitro, PDIA4 knockdown not only increased apoptosis of tumor cells with/without radiotherapy, but also decreased the ability of proliferation. In contrast, overexpression of PDIA4 induced the opposite effects on apoptosis and proliferation. According to Co-IP/MS results, PDIA4 prevented Tax1 binding protein 1 (TAX1BP1) degradation by binding to TAX1BP1, which inhibited c-Jun N-terminal kinase (JNK) activation. Moreover, PDIA4 knockdown suppressed tumor growth xenograft model in vivo, which was accompanied by an increase in apoptosis and promoted tumor growth inhibition after radiotherapy.

**Conclusions:**

The results of this study indicate that PDIA4 is an oncoprotein that promotes TNBC progression, and targeted therapy may represent a new and effective anti-tumor strategy, especially for patients with radiotherapy resistance.

**Supplementary Information:**

The online version contains supplementary material available at 10.1186/s13058-023-01758-6.

## Introduction

According to a report released by the World Health Organization in 2020, the number of breast cancer (BC) cases is on the rise worldwide. It is estimated that there are 2.3 million new breast cancer cases worldwide, accounting for 11.7% of the world’s new cases. As the malignant tumor with the largest number of cases in the world, breast cancer surpassed lung cancer for the first time, with 18.4% of the global new cases occurring in China [1–3]. Breast cancer is treated in a variety of ways, including surgery, radiotherapy, chemotherapy, endocrine therapy and molecular targeted therapy. As the most aggressive subtype of breast cancer, triple-negative breast cancer is characterized by the absence of estrogen, progesterone, and human epidermal growth factor receptor type 2, it is associated with a poor prognosis, a high risk of recurrence, and a high risk of metastasis [4–6]. Radiotherapy (RT) is one of the methods used to treat malignant tumors and metastatic tumors on a local level. RT causes irreversible damage to the DNA of cancer cells in the irradiation field, thereby causing cancer cells to die through apoptosis, necrosis, autophagy and so on [7–9]. As a result of RT, tumor-associated antigens (TAAs) are released, cytokines are produced, the tumor microenvironment is altered, and the body’s immune system is activated in an effort to assist in the immune response against tumors [8, 10, 11]. RT is the standard treatment for locally advanced breast cancer. Combined with breast conserving surgery, adjuvant RT reduces the risk of the first recurrence from 35.0 to 19.3% and from 25.2 to 21.4% over the next 15 years [12]. Adjuvant RT, however, can result in local recurrences in some patients. A 5-year local recurrence rate for adjuvant RT following breast conserving surgery is about 2.7%, while a 5-year local recurrence rate for adjuvant RT following modified radical surgery is approximately 6.1% [13]. Postoperative RT can improve local control, especially in TNBC [14]. Inherent or acquired RT resistance is the most important factor affecting local recurrence, which is very similar to chemotherapy resistance, which results in poor treatment effects, rapid tumor progression, malignant invasion, and ultimately failure of RT in these patients [15]. Additionally, multiple factors affect cancer RT resistance mechanisms. Thus, identifying RT resistance biomarkers and elucidating underlying biological mechanisms is essential for identifying clinical approaches that can improve response to RT.

The JNK signaling pathway can be activated by various extracellular stimuli, such as tumor necrosis factor-alpha, interleukin-1, epidermal growth factor, certain G protein coupled receptor, stress (such as ionizing radiation, osmotic pressure, heat shock, and oxidative damage). It is involved in many biological reactions such as cell proliferation and differentiation, cell morphology maintenance, cytoskeleton construction, cell apoptosis and cell malignant transformation [16, 17]. The cause of radiation-induced cell death is DNA damage, either through direct ionization of DNA or indirectly through the generation of reactive oxygen species (ROS) [18–20]. It is possible for RT to directly damage DNA and indirectly damage DNA via ROS generated upon radioactive dissolution of water, the primary constituent of intracellular fluid. Direct and indirect damage to DNA result in DNA damage response. Radiation-induced DNA damage response can result in DNA repair, G2/M arrest, and apoptosis, which ultimately leads to death [21, 22]. It has been demonstrated recently that ionizing radiation-related stress promotes endoplasmic reticulum stress. When IRE1 is activated for a prolonged period of time, it will interact with tumor necrosis factor receptor-related factor-2, which will lead to the activation of JNK, which will lead to downstream responses and ultimately induce apoptosis [23–26].

It is activated by a variety of extracellular stimuli, including cell proliferation and differentiation, cell morphology maintenance, cytoskeleton construction, cell apoptosis and cell malignancy. Many previous studies have suggested that the JNK pathway may play an important role in resistance to RT [27–29].

c-Jun is the prototype substrate of JNK and an important part of JNK pathway, which can indirectly reflect the activity of JNK. c-Jun plays an important role in the activation of activator protein 1 (AP-1) complex and plays an important role in the transformation process of cell proliferation and differentiation. JNK binds and phosphorylates c-Jun and increases its transcriptional activity, thereby activating an important component of the AP-1 transcription complex, which is an important regulator of gene expression [30–32].

Protein disulfide isomerase family member 4 (PDIA4) is a member of protein disulfide isomerase (PDI) family, which is located primarily in the endoplasmic reticulum (ER). It contains 645 amino acids and three classical CGHC active motifs. It is induced by ER stress and initiates clotting and enhances thrombosis by acting as an oxidoreductase [33, 34]. There is increasing evidence that PDI plays a role in cancer survival and progression through its regulation of ER stress, apoptosis, DNA repair, and autophagy, by inhibiting autophagy through its thioredoxin 6 domain, and PDI enhances the resistance of tumor cells to radiotherapy and chemotherapy by decreasing autophagy through the ERS pathway [35, 36]. GBM cells with PDI knockout and ionizing radiation (IR) have increased cytotoxicity, associated with a reduced ability to repair IR-induced DNA damage [37]. Several studies have shown that PDIA4 is overexpressed in human malignancies, including esophageal squamous cell carcinoma, and high levels of PDIA4 expression were associated with poor glioma survival rates [38, 39]. It was revealed that modulating PDIA4 expression in cancer cells allowed the cells to grow by regulating caspase 3 and 7, docetaxel resistance was induced by PDIA4 activation and inhibited prostate cancer cell apoptosis, while PDIA4 inactivation restored classical mitochondrial apoptosis [40–42]. However, the role and mechanism of PDIA4 in RT resistance of TNBC have not been reported yet.

Here, PDIA4 expression is significantly higher in TNBC tumor tissues than in normal tissues adjacent to the tumor. In addition, after PDIA4 knockdown or overexpression, the clonogenic ability of TNBC cells after RT was significantly lower or higher than that of the control group. The expression of PDIA4 in TNBC cell lines was significantly negatively correlated with apoptosis and radiotherapy sensitivity. Xenograft model was also used to validate these phenotypes. Co-IP/MS results suggest that PDIA4 could prevent TAX1BP1 degradation when combined with TAX1BP1, whereas excessive TAX1BP1 inhibits JNK activation and apoptosis. We conducted this study to examine whether PDIA4 is a RT resistance biomarker in TNBC cells as well as to characterize the mechanisms that underlie it.

## Materials and methods

### Bioinformatics

We use the cancer genome atlas (TCGA) database (https://genome-cancer.ucsc.edu/) to predict PDIA4 mRNA expression in BC and TNBC and its relationship with overall survival (OS) of TNBC patients. The UALCAN database (https://ualcan.path.uab.edu/analysis.html) was used to analyze PDIA4 expression in various types of breast cancer. GEPIA database (http://gepia.cancerpku.cn/) was used to generic cancer PDIA4 gene analysis.

### Tissue specimens

A total of 30 paired TNBC samples were obtained from surgical resections performed at the Department of Breast Surgery, Drum Tower Hospital Affiliated to Nanjing University School of Medicine (Nanjing, China), between 2018 and 2022. All patients provided written informed consent for the study. In accordance with health insurance portability and accountability act (HIPAA) guidelines and institutional protocols, tissue specimens were frozen in liquid nitrogen and stored at − 80 °C prior to use. Each specimen was examined and analyzed by two experienced pathologists.

### Cell culture and the development of stable cell lines

The MDA-MB-231 and MDA-MB-468 breast cancer cell lines, as well as HEK293, were obtained from the American Technical Ceramics Corporation (ATCC, Manassas, USA). Cells were cultured at 37 °C in 5% CO_2_ in DMEM supplemented with 10% fetal bovine serum and 1% penicillin/streptomycin. Infected cells were incubated with specific lentiviral vectors for 48 h and selected for 2 weeks with puromycin. No evidence of mycoplasma contamination was detected in any of the cell lines.

### Plasmid construction

Encoding PDIA4, TAX1BP1 and short hairpin RNA (shRNA), including a nonsense sequence shNC and against PDIA4 sequences shRNA1, shRNA2 and shRNA3 (Additional file 1: Table S1), were designed and synthesized by the Corues Biotechnology Company (Nanjing, China).

### Reagents and antibodies

Anti‐PDIA4 (14,712–1-AP) and anti‐TAX1BP1 (14,424–1-AP) antibodies were purchased from Proteintech Group Inc (Wuhan, China); anti‐Bcl‐2 (15,071), anti‐Bax (41,162), anti‐cleaved-PARP1 (5625), anti‐p‐JNK (4668), anti‐JNK (67,096), anti‐β‐actin (3700), anti-mouse IgG HRP (93,702), and anti‐rabbit IgG HRP (14,708) antibodies were purchased from Cell Signaling Technology Inc. (Massachusetts, USA); anti‐c‐Jun (sc-166540) and anti‐p‐c‐Jun (sc-822) antibodies were purchased from Santa Cruz Biotechnology Inc. Anti‐TAX1BP1 (ab245636) was purchased from Abcam Inc. (Massachusetts, USA). The reagents used included MG-132 (HY-13259), cycloheximide (CHX) (HY-12320) and SP600125 (HY-12041) which were obtained from MedChemExpress (Shanghai, China) and TRIzol obtained from Invitrogen (California, USA). Dulbecco’s modified Eagle’s medium (DMEM) and fetal bovine serum were purchased from Gibco (Massachusetts, USA).

### Radiation

Using the RS2000 Pro225 X-ray biological irradiator (Georgia, USA) irradiate the target cells at room temperature with different irradiation doses.

#### Cell proliferation assay and clonogenic assay

Approximately 2,000 cells, which were untreated or treated with 4 Gy of radiation once, were inoculated into each well of a 96-well plate, and each hole’s optical density was measured daily at 450 nm by Cell Counting Kit-8 (CCK-8) (Vazyme, Nanjing, China). Cells were seeded into 6-well plates, 1000 cells per well, which were exposed to 0, 2, 4, 6 and 8 Gy of radiation once. After 14 days of culture in complete medium, colonies appeared and were stained with crystal violet. The number of colonies was recorded.

### Reverse transcription and real-time PCR

A total of RNA was obtained using TRIzol reagent (Invitrogen, California, USA). Reverse transcription was performed using the RNA Reverse Transcription Kit (Vazyme, Nanjing, China). The RT-qPCR was performed with TaqMan PCR mix (Vazyme, Nanjing, China) according to standard protocols and the cycle time (Ct) of selected genes was normalized to GAPDH expression. The relative expression of each gene was calculated using the 2^−ΔCt^ method. Primer sequences used for amplification were listed in Additional file 2: Table S2.

### Western blot

The total protein of cells and tissues is extracted using protein lysate (Beyotime, Shanghai, China), separated by electrophoresis on a 10–15% SDS polyacrylamide gel, then transferred to a polyvinylidene fluoride membrane (PVDF). Blocking was performed in Tris-buffered saline and Tween-20 (TBST) containing 5% skim milk for 2 h, followed by incubation with the primary antibody at 4 °C overnight and with the secondary antibody at 4 °C for 2 h. An enhanced chemiluminescence detection system was used to detect signals with a chemiluminescent HRP substrate. β-actin was used as an internal control.

### Flow cytometry

The apoptosis of apoptotic cells was assessed by flow cytometry. 2 × 10^5^ cells were seeded overnight in 6-well plates and then treated with IR for 24 h. Thereafter, the cells were collected, washed three times with cold PBS, stained with Annexin V-PE and 7-AAD (Vazyme, Nanjing, China) in binding buffer for 10 min at room temperature and in the dark and then quantified with a BD FACSCalibur flow cytometer (BD Sciences, USA).

### Immunoprecipitation coupled with mass spectrometry (IP/MS)

Proteins were extracted from breast cancer cells and analyzed using the indicated primary antibodies and protein A/G agarose beads (Beyotime, Shanghai, China). Separated immunoprecipitates were then analyzed by mass spectrometry (Applied Protein Technology).

### Co-immunoprecipitation assay (co-IP)

We conducted a co-IP analysis to verify the interaction between PDIA4 and TAX1BP1, and immunoprecipitated proteins from cell lysates were detected by Western blotting using protein A/G agarose beads, following the manufacturer’s instructions.

### Immunohistochemistry assays (IHC)

For immunohistochemical staining, the specimens were fixed in 4% formalin and embedded in paraffin blocks, which were then cut into 4 μm sections and incubated overnight at 4 °C with anti-PDIA4. Afterward, the sections were rinsed three times with PBS and incubated for 2 h at 37 °C with the conjugated HRP polymer and secondary antibody. After counterstaining with 3,3′-diaminobenzidine solution for 3 min, counterstaining with hematoxylin, the samples were examined by a microscope in a blinded manner. Images were obtained from three random fields for each sample.

### Immunofluorescence (IF)

For immunohistochemical staining, the specimens were fixed in 4% formalin and embedded in paraffin blocks, which were then cut into 4 μm sections and incubated overnight at 4 °C with anti-PDIA4 and anti-TAX1BP1. Fluorescent or biotin-labeled secondary antibodies were added to the tissues to observe the protein expression.

### Mitochondrial membrane potential (MMP) detection with MitoTracker Red CMXRos (TRC) staining

The intracellular *MMP* levels were detected using the TRC obtained from Beyotime Biotech (Shanghai, China) according to the manufacturer’s instructions. Cells were washed three times with PBS and incubated with serum-free DMEM medium containing TRC for 20 min in the dark at 37 °C. Cells were then stained with Hoechst 33,342 for approximately 5–10 min. Observe under a confocal microscope following three washes in PBS.

### Animal experiment

The experiments on animals were conducted in accordance with the guidelines for the care and use of experimental animals approved by the Ethics Committee of Drum Tower Hospital Affiliated to Nanjing University School of Medicine. Female BALB/c nude mice were obtained from Gempharmatech Co., Ltd (Nanjing, China). Mice were injected with control cells (shNC) or PDIA4 knockdown cells (shRNA3) of MDA-MB-231cell line (1 × 10^6^) subcutaneously in the right thigh and tumors were allowed to grow. Once the tumor volume reached 70–100 mm^3^, the mice were separated into different groups and each group consists of five mice. It was determined that a fractionated IR dose of 5 × 1 Gy would simulate the classical regimen used to treat one week of patients for radiation. Every 4 days, the tumor volume was measured using calipers with a two-dimensional plane. We sacrificed mice four weeks after injection and calculated tumor volume by using the formula (width^2^ × length)/2. Tumor tissue was then collected for immunostaining.

### Terminal deoxynucleotidyl transferase dUTP nick end labeling assays (TUNEL)

The tissues were deparaffinized, washed in absolute ethanol for 5 min, rehydrated in decreasing concentrations of ethanol, washed twice in PBS, and incubated with proteinase K solution for 30 min. Apoptosis was then detected by TUNEL staining (TUNEL BrightRed Apoptosis Detection Kit from Vazyme) according to the manufacturer’s instructions.

### Statistical analysis

In this study, the data were calculated based on the results of at least three independent experiments carried out using GraphPad Prism 9.0.0 (GraphPad Software, USA) and SPSS (IBM Software, USA) version 26.0 (IBM, USA). Differences between groups were assessed with a two-tailed Student’s t test, an analysis of variance (ANOVA) or χ^2^ test. Statistical significance was defined as a *P* value less than 0.05.

## Results

### PDIA4 expression was upregulated in BC and TNBC patients and associated with the survival of TNBC patients

**Fig. 1:**
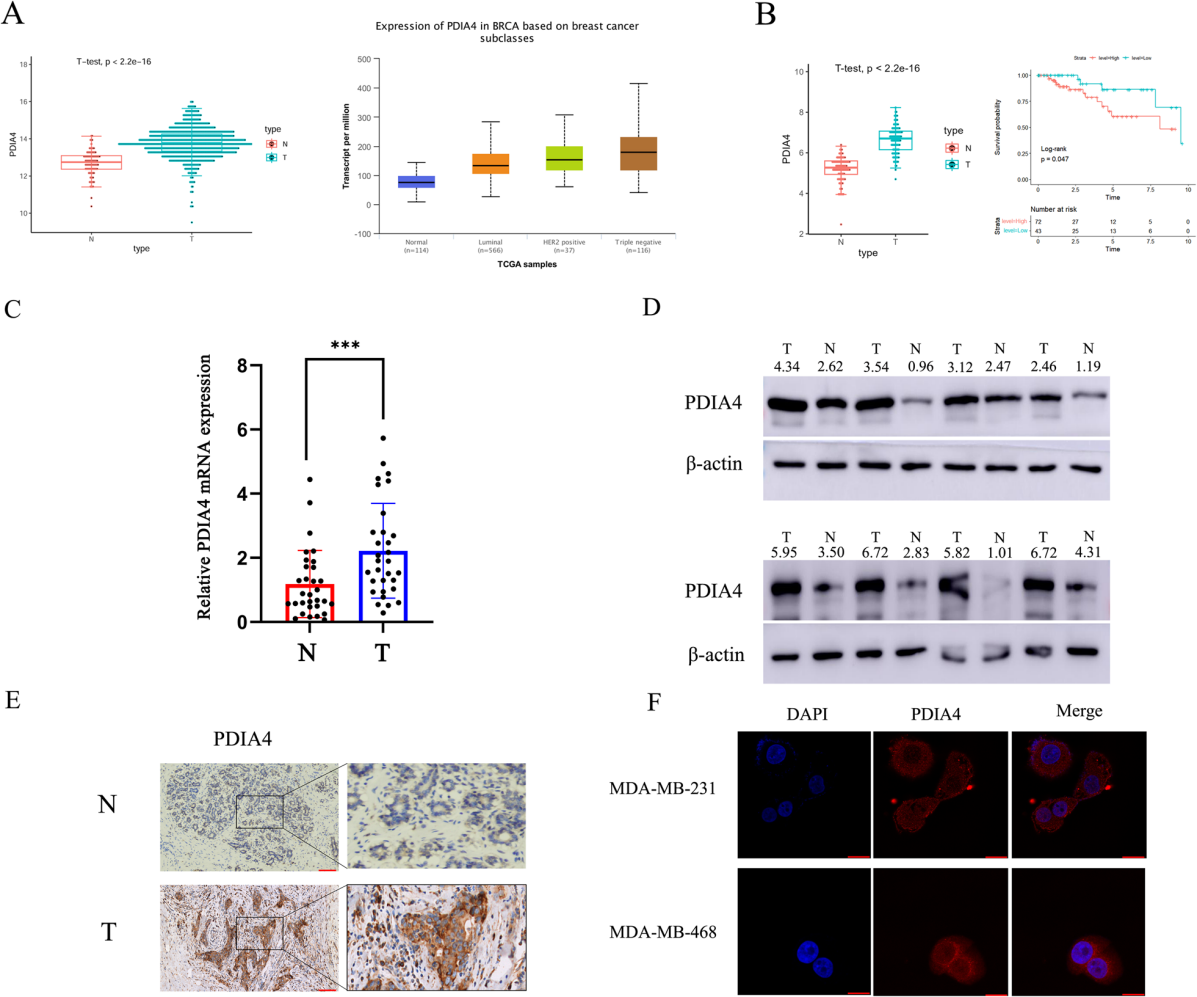
PDIA4 was upregulated in BC and TNBC and associated with TNBC progression and prognosis. **A** PDIA4 mRNA was significantly higher in BC tissues than the adjacent normal tissues based on the TCGA dataset (1105 BC tissues and 113 adjacent normal tissues). In a subclass analysis of breast cancer, PDIA4 mRNA expression was significantly higher in TNBC than in luminal and Her-2 positive cancers in UALCAN database. **B** PDIA4 mRNA was significantly higher in TNBC tissues than the adjacent normal tissues based on the TCGA dataset (116 TNBC tissues and 113 adjacent normal tissues). Kaplan–Meier survival analysis showed that higher PDIA4 expression was associated with poor OS in TNBC patients. **C** The expression level of PDIA4 in TNBC and adjacent non-tumor tissues was analyzed by RT-PCR. **D** The expression levels of PDIA4 in eight pairs of TNBC tissues (T) and adjacent non-tumor tissues (N) were analyzed by western blot. **E** Representative results of the upregulation of PDIA4 protein in TNBC specimens via immunohistochemistry. **F** Representative results of the upregulation of PDIA4 protein in TNBC specimens via immunofluorescence. All data are shown as the mean ± S.D. of at least three independent experiments. The *P* values were calculated using unpaired Student’s t test. *P < 0.05; ***P < 0.001

On the basis of the TCGA dataset (1105 BC tissues and 113 adjacent normal tissues), we assessed the expression of PDIA4 in BC and its clinical significance. Overall, the expression of PDIA4 mRNA was significantly higher in BC tissues than in adjacent normal tissues (*P* < 0.001). In a subclass analysis of breast cancer, PDIA4 mRNA expression was significantly higher in TNBC than in luminal and Her-2 positive cancers in UALCAN database (Fig. 1A). PDIA4 mRNA was significantly higher in TNBC tissues than the adjacent normal tissues based on the TCGA dataset (116 TNBC tissues and 113 adjacent normal tissues). Further, survival analysis revealed that patients with TNBC who expressed high levels of PDIA4 had a lower overall survival (OS) (Fig. 1B). Also pan-cancer analysis of PDIA4 was performed utilizing the GEPIA2 database (Additional file 3: Fig.S1). To verify the bioinformatics results, PDIA4 mRNA expression levels were analyzed by RT-PCR in 30 matched tumor tissues and adjacent tissues. Results showed that tumor tissues expressed significantly more PDIA4 mRNA than adjacent tissues (Fig. 1C). Furthermore, we performed western blots on 10 paired breast cancer tissues and normal tissues to examine the protein level of PDIA4 (Fig. 1D). A comparison of the expression levels of PDIA4 protein in TNBC tissues and paracancerous tissues was also conducted by IHC and IF, which demonstrated a higher expression level of PDIA4 protein in TNBC tissues but a lower expression level in paracancerous tissues (Fig. 1E and F).

### PDIA4 promoted cancer properties of TNBC in vitro

**Fig. 2:**
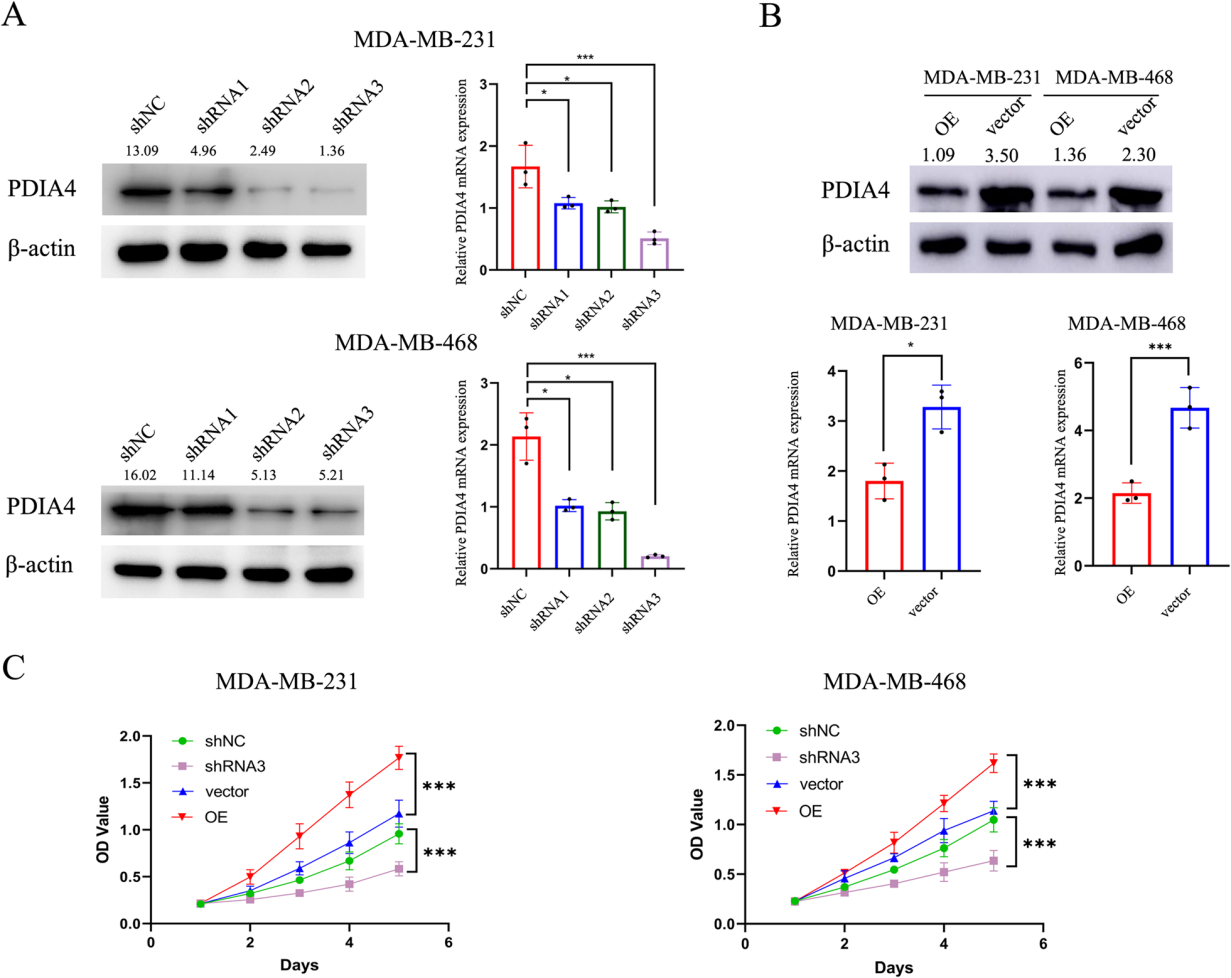
PDIA4 promoted cell proliferation in TNBC. **A** PDIA4 knockdown TNBC cells was constructed by infecting MDA-MB-231 and MDA-MB-468 cells with lentivirus, and the expression efficiency of the cells was assessed through western blot and RT-PCR. **B** Lentivirus transfected TNBC cells with PDIA4 overexpression, and western blot and RT-PCR were used to detect the efficiency. **C** The effects of PDIA4 on TNBC cells proliferation were assessed using the CCK-8 assay. Results indicated that overexpression of PDIA4 could promote cell proliferation, while downregulation of PDIA4 was able to significantly inhibit cell proliferation. All data are shown as the mean ± S.D. of at least three independent experiments. The *P* values were calculated using unpaired Student’s t test. *P < 0.05; ***P < 0.001

Transfection of shRNA by lentivirus inhibited PDIA4 expression. We assessed the knockdown efficiency of the three PDIA4-specific shRNAs by RT-qPCR and western blot, respectively, and compared them to the negative control. The results showed that shRNA3 mediated the knockdown efficiency with the highest efficiency, so it was selected for further investigation (Fig. 2A). Transfecting TNBC cells with LV-PDIA4 stably overexpressed PDIA4, and based on RT-qPCR and western blot analysis, PDIA4 was overexpressed at the mRNA and protein levels, respectively, compared with the negative control (Fig. 2B). We then observed that downregulation of PDIA4 led to significant inhibition of cell proliferation, while overexpression of PDIA4 promoted cell proliferation as measured by CCK-8 assays (Fig. 2C). Flow cytometry (FACS) assay showed that PDIA4 silencing could significantly increase the apoptosis rate. In contrast, the opposite result was observed after overexpression of PDIA4 in TNBC cells (Fig. 4A and B).

### PDIA4 is closely related to the resistance of TNBC to radiation

**Fig. 3:**
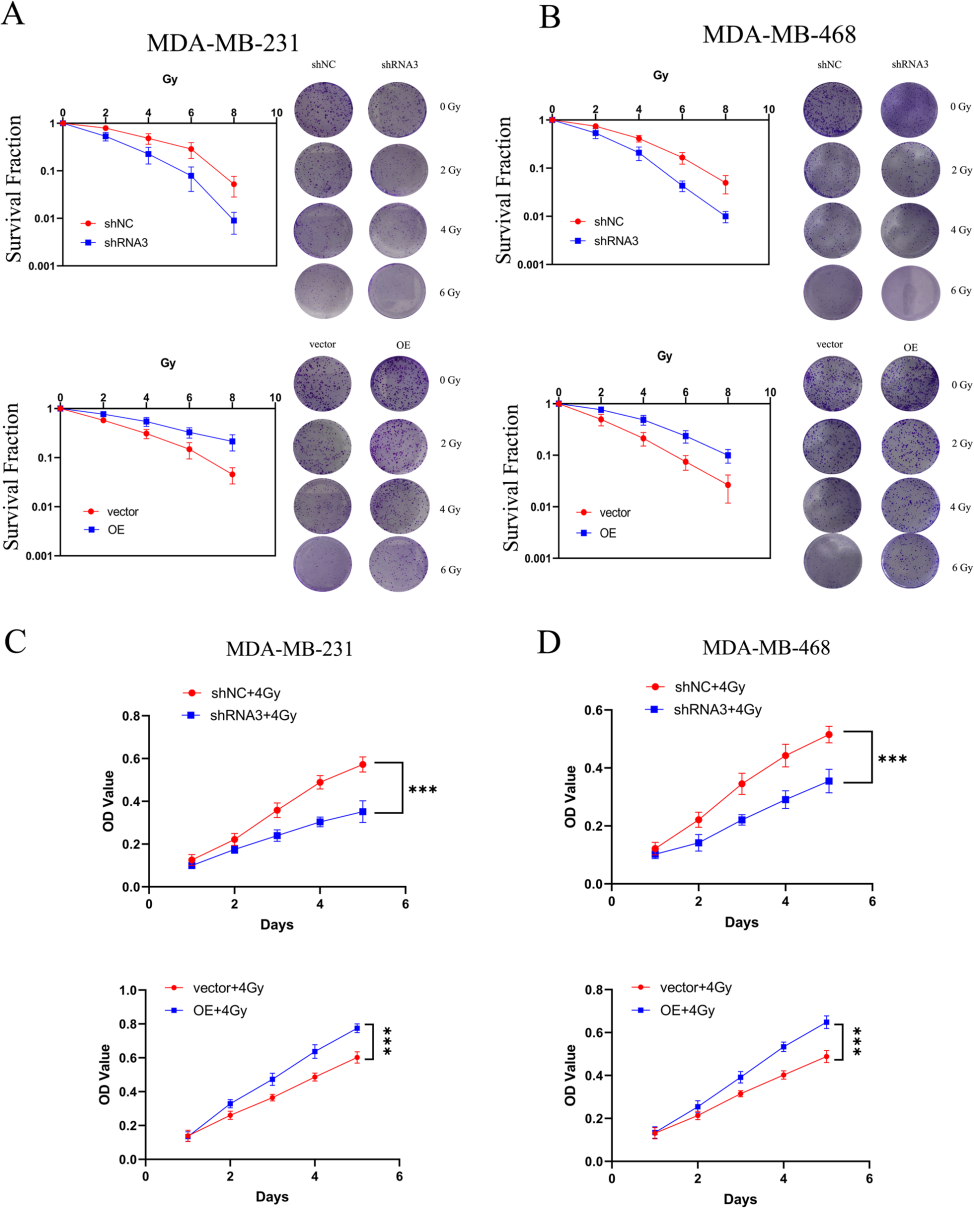
PDIA4 induces proliferative effects in TNBC cells with/without IR. **A** and **B** Surviving fraction of MDA-MB-231 and MDA-MB-468 cells treated with a single dose of radiation (0–8 Gy) after downregulation of PDIA4 or overexpression of PDIA4, measured 2 weeks with/without IR. **C** and **D** CCK-8 assay was used to assess the proliferation of knockdown or overexpression of PDIA4 TNBC cells with/without IR. All data are shown as the mean ± S.D. of at least three independent experiments. The *P* values were calculated using unpaired Student’s t test. *P < 0.05; ***P < 0.001

**Fig. 4:**
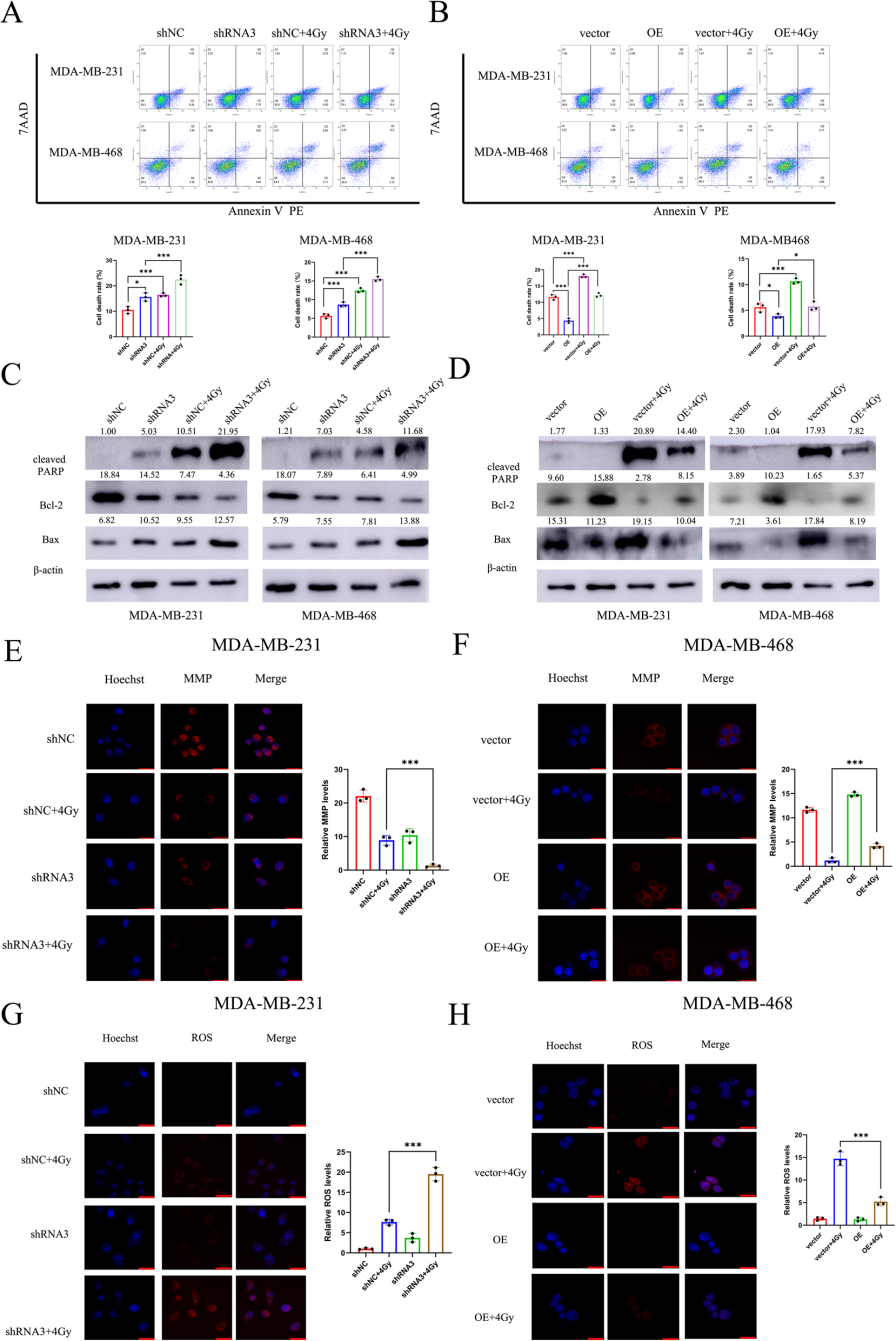
PDIA4 inhibited apoptosis effects in TNBC cells with/without IR. **A** and **B** Flow cytometric analysis of apoptosis in MDA-MB-231 and MDA-MB-468 cells infected with lentivirus PDIA4 or shRNA3 with/without IR. **C** and **D** Western blot analysis showed that knockdown of PDIA4 increased the levels of cleaved-PARP and Bax in TNBC cells while reducing the expression level of Bcl-2 with/without IR and the opposite results were observed when PDIA4 was overexpression in TNBC cells with/without IR. **E** and **F** MMP was analyzed using immunofluorescence in TNBC cells of knockdown or overexpressing of PDIA4 with/without IR. **G** and **H** ROS was analyzed using immunofluorescence in TNBC cells of knockdown or overexpressing of PDIA4 with/without IR

PDIA4 knockdown had lower colony-forming ability in MDA-MB-231 and MDA-MB-468 cells, and the same results as above were obtained from the CCK-8 cell viability assay with/without IR (Fig. 3A and 3C), which provided a further insight into the involvement of PDIA4 in RT resistance in vitro, whereas the overexpression of PDIA4 had the opposite effect (Fig. 4B and D).

The apoptosis rates of MDA-MB-231 and MDA-MB-468 cells after PDIA4 gene knockdown and IR were measured by flow cytometry. The apoptosis rate of MDA-MB-231 and MDA-MB-468 cells treated with PDIA4 knockdown or controls and then IR was determined using FACS analysis, the results showed that knockdown of PDIA4 expression was significantly higher than that of control cells after IR, and the opposite results were observed in upregulated in PDIA4-overexpressing TNBC cells (Fig. 4A and B). Additionally, we used western blot to detect the expression of cleaved-PARP, Bcl-2 and Bax, the results showed that cleaved-PARP and Bax levels were significantly higher in MDA-MB-231 and MDA-MB-468 cells of PDIA4 knockdown, whereas Bcl-2 levels were the opposite after IR, in contrast, when PDIA4 was overexpressed in the TNBC cells, cleaved-PARP and Bax levels were downregulated, whereas Bcl-2 levels was significantly higher than vector after IR (Fig. 4C and D). These results indicate that PDIA4 plays an important role in RT resistance of TNBC cells.

TRC and DHE staining was used to detect MMP and ROS. Under IR treatment, the red fluorescence in cells with PDIA4 knockdown was significantly reduced compared to the control group, whereas PDIA4 was overexpressed MMP was higher than vector (Fig. 4E and F). On the contrary, ROS levels were higher in PDIA4 downregulated of TNBC cells than in shNC after IR, while ROS levels were lower in PDIA4-overexpressed cells (Fig. 4G and H). The results showed that MMP decreased and ROS increased, mitochondria were damaged and apoptosis increased.

### Loss of PDIA4 promotes radiosensitivity and inhibits tumorigenesis in vivo

**Fig. 5:**
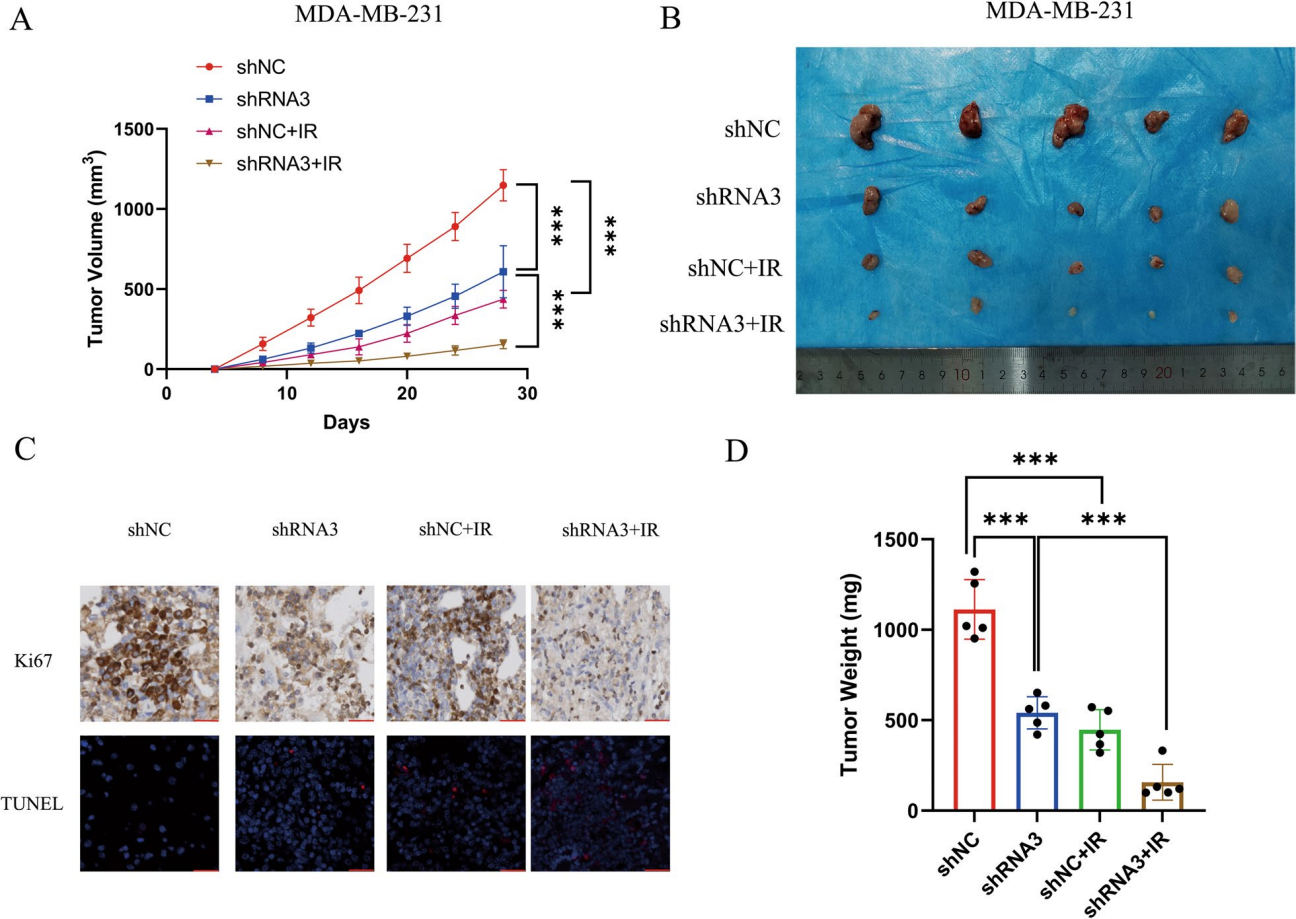
PDIA4 knockdown induced sensitivity of RT in murine xenografts. **A** Tumor volume was measured at the indicated days with/without IR (two-way ANOVA; *n* = 5; *, *P* < 0.05; ***, *P* < 0.001). **B** Photograph of the dissected tumors. **C** Immunohistochemical staining (Ki67) and TUNEL staining of PDIA4 xenograft tumor sections with/without IR. **D** Tumor weight was measured in the indicated groups with/without IR (t test; *n* = 5; *, *P* < 0.05; ***, *P* < 0.001)

In view of the fact that knockdown of PDIA4 expression increased the sensitivity of TNBC to radiation in vitro, we next examined the effect of knockdown of PDIA4 expression in mice. For this purpose, the stable MDA-MB-231 cell line with PDIA4 expression knockdown was subcutaneously injected into nude mice to construct a xenograft tumor mouse model. We then irradiated mice with 5 Gy. Tumor size was measured every 4 days. After 4 weeks, mice were sacrificed and tumors were excised and weighed. The effects of various treatments on tumor volume and appearance are shown in Fig. 5A and B. We found that tumor weight was the lightest observed in the PDIA4 shRNA3 + IR group, with significant differences (Fig. 5D). In addition, the TUNEL was used to measure the apoptosis of tumor histiocyte in vivo. The apoptosis rate of cells in the PDIA4 shRNA3 + IR treated group was significantly higher than that in the control group, and the immunohistochemical results of Ki67 were opposite to those of TUNEL (Fig. 5C). These data indicate that PDIA4 knockdown can inhibit tumor growth in TNBC and enhance the sensitivity of RT.

### PDIA4 interacted with TAX1BP1 and inhibited proteasomal degradation of TAX1BP1

**Fig. 6:**
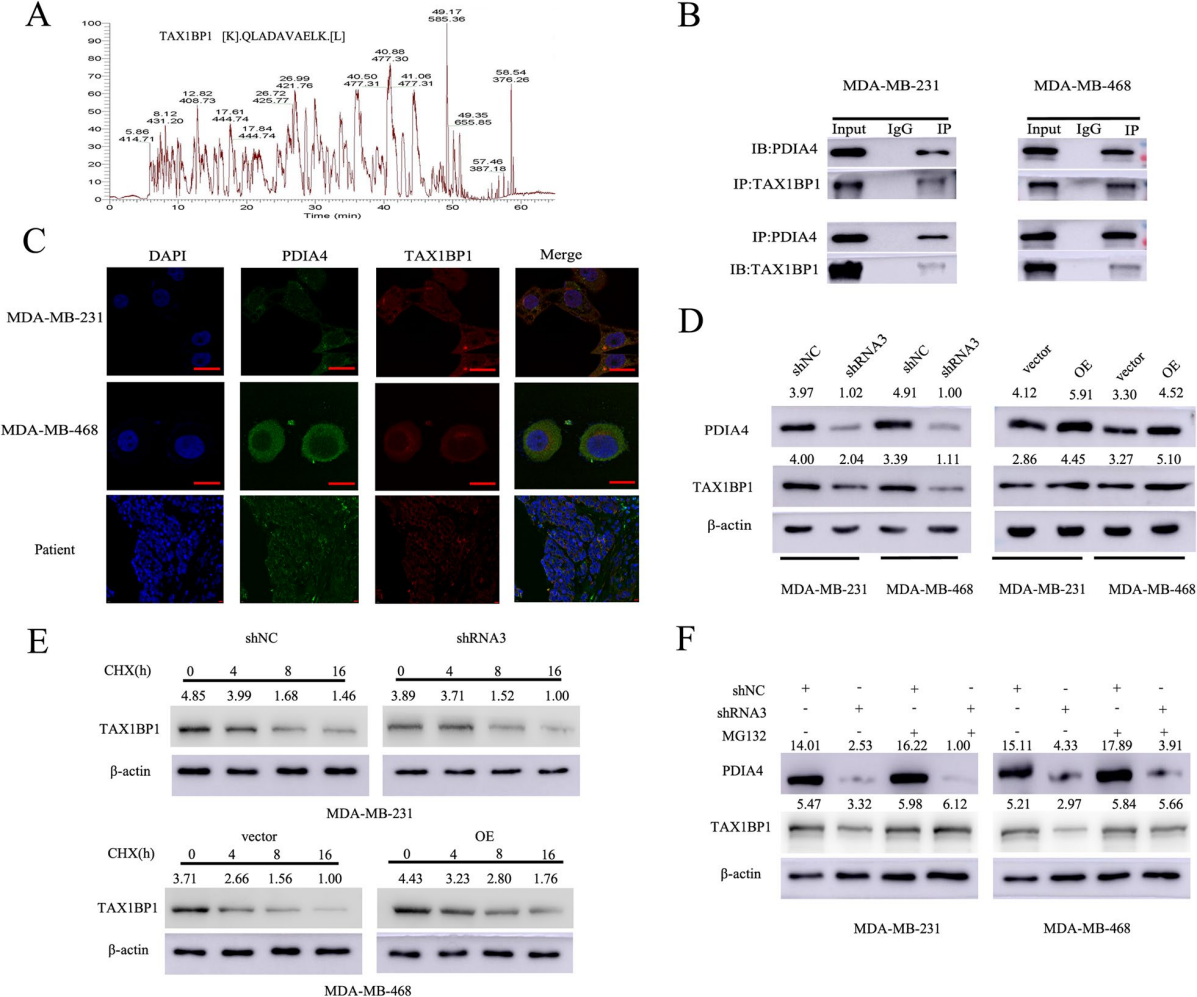
PDIA4 promoted tumorigenesis and RT resistance by binding and protecting TAX1BP1 against protein degradation. **A** PDIA4 can bind TAX1BP1 with a high binding fraction as determined by IP/MS analysis. **B** Co-IP experiments demonstrated that PDIA4 immunoprecipitated with anti-TAX1BP1 antibody, and TAX1BP1 immunoprecipitated with anti-PDIA4 antibody. **C** Immunofluorescence was conducted to examine the expression and localization of PDIA4 and TAX1BP1, the results showed that PDIA4 and TAX1BP1 was localized mainly in the cytoplasm of TNBC cells and cancer specimens. **D** Western blot analysis of TAX1BP1 expression levels in TNBC cells with PDIA4 knockdown or overexpression. **E** Western blot analysis of TAX1BP1 expression levels in TNBC cells treated with CHX for the indicated times. **F** MDA-MB-231 and MDA-MB-468 cells were treated with/without MG132, and TAX1BP1 protein levels were detected by western blot

In order to explore the mechanisms which PDIA4 regulates tumorigenesis and RT resistance, IP/MS was used to identify proteins binding to PDIA4, as shown in Fig. 6A, and TAX1BP1 may be a protein that interacts with PDIA4. It is well-known that TAX1BP1 plays an important role in the regulation of various physiological and pathological phenomena, such as cell apoptosis, embryonic development and immunity through NF-kB and JNK signaling pathways, but it is also widely observed that abnormal expression of this protein is related to inflammation, malignant tumors, circulatory system diseases, etc. [43–45]. The role of PDIA4 in regulating tumorigenesis and radiation resistance of TNBC is unclear. Co-IP of PDIA4-binding proteins provides an understanding of this role. An antibody specific for PDIA4 co-immunoprecipitated TAX1BP1, while an antibody specific for TAX1BP1 co-immunoprecipitated PDIA4, according to our findings, PDIA4 acted as a binding partner for TAX1BP1 in TNBC cells and cancer specimens (Fig. 6B and C). PDIA4 knockdown led to reduced TAX1BP1 protein expression in TNBC cells. Conversely, ectopic PDIA4 expression increased TAX1BP1 protein expression (Fig. 6D). The next step was to determine whether PDIA4 affects the stability of TAX1BP1 by using CHX to determine the protein stability of TAX1BP1. The results showed that TAX1BP1 was more stable when PDIA4 was highly expressed and vice versa (Fig. 6E). In addition, we cultured MDA-MB-231 and MDA-MB-468 cells with MG132 which is supposed to repress the proteasomal degradation pathway to prevent protein degradation. MG132 significantly restored the expression of TAX1BP1 in PDIA4-silenced cells (Fig. 6F), suggesting that PDIA4 inhibits the proteasome of TAX1BP1 degradation.

### The PDIA4 regulates RT resistance in TNBC cells by the JNK/c-Jun signaling pathway through TAX1BP1

**Fig. 7:**
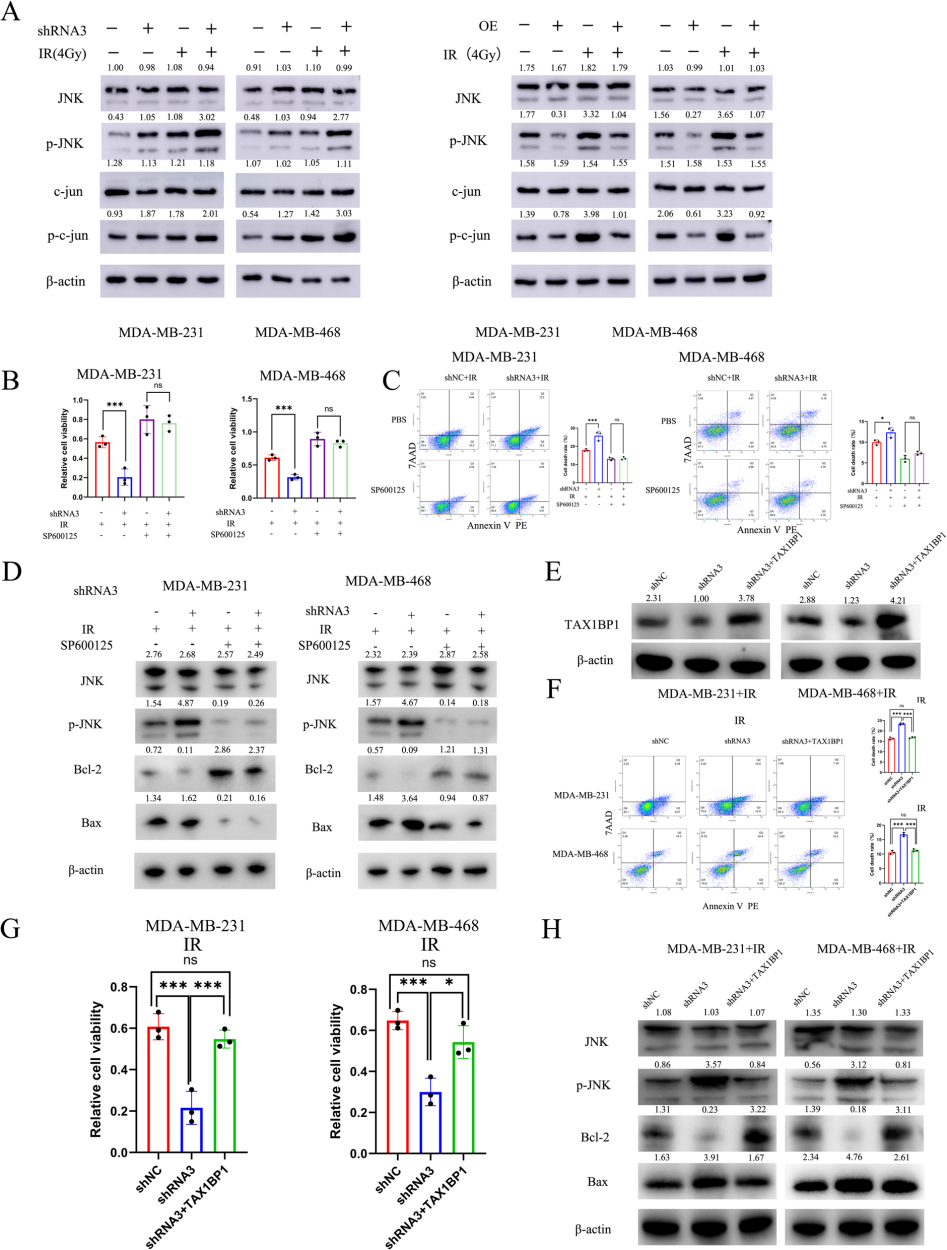
PDAI4 via JNK/c‐Jun signaling to enhance tumorigenesis and RT resistance. **A** Western blot analysis of JNK/c‐Jun signaling pathway‐related proteins, the results revealed that JNK, c‐Jun, p‐JNK, p‐c‐Jun expression levels in TNBC cells with PDIA4 knockdown or overexpression with/without IR. **B**, **C** and **D** The target cells were transfected with shRNA3 or shNC with/without IR, then cultured with SP600125, cell proliferation was tested using the CCK‐8 assay, apoptosis was detected by FACS assay, and the protein was analyzed by western blot. **E** Western blot analysis of TAX1BP1 expression after transfection. **F**, **G** and **H** The target cells with or without TAX1BP1 overexpression following treatment with IR, cell proliferation was tested using the CCK‐8 assay, apoptosis was detected by FACS assay, and the protein was analyzed by western blot

It has been shown that the JNK signaling pathway plays an important role in tumor cell drug and RT resistance [46, 47]. We detected JNK pathway-related proteins by western blot, and the results indicated that p-JNK and p–c-Jun were significantly increased in irradiated TNBC cells with PDIA4 knockdown, while total expression levels of JNK and c-Jun were respectively little changed and vice versa (Fig. 7A). SP600125 is a commonly used inhibitor of the JNK pathway that is highly selective [48], in order to further evaluate the effects of PDIA4 on the JNK signaling pathway, target cells were treated with SP600125 and their viability, apoptosis, and western blot were assessed (Fig. 7B, C and D). The results indicated that SP600125 inhibited PDIA4-induced JNK activation and PDIA4-induced apoptosis with IR. Furthermore, we investigated whether altered TAX1BP1 expression could contribute to PDIA4-induced RT resistance progression in TNBC cells through the induction of TAX1BP1 overexpression. Results indicate that overexpression of TAX1BP1 can reverse the decreased ability of RT resistance of TNBC cells following knockdown of PDIA4 (Fig. 7E, F, G and H). These observations suggested that PDIA4 may affect tumorigenesis and RT resistance through TAX1BP1/JNK/c-Jun signaling.

## Discussion

Estrogen receptor (ER), progesterone receptor (PR) and human epidermal growth factor receptor 2 (Her-2) are unactivated in TNBC [49]. TNBC is a form of breast cancer that exhibits special molecular and biological characteristics, which is often associated with young age at onset, a high malignant biological behavior, easy metastasis and poor prognosis, approximately 10–20% of all breast cancer patients are affected by this disease [50, 51]. In spite of improvements in the overall survival rate of breast cancer, 30–40% of patients of TNBC still experience uncontrollable local recurrence and distant metastases. RT can effectively improve the local control rate and decrease distant metastases caused by uncontrolled local disease, as one of the main means of comprehensive tumor treatment [52–54]. However, TNBC is the presence of inherent and/or acquired RT resistance, which is an important reason for RT failure. Recent studies indicate that many cellular molecular targets are involved in the RT resistance of TNBC [55–58]. Therefore, it is imperative that new strategies be explored to combat RT resistance of TNBC.

One of the main mechanisms of radiation killing tumor cells is the induction of apoptosis in tumor cells, which is mainly mediated through signaling pathways controlled by the mitochondria [59–62]. Apoptosis is one of the most important factors in cell life and could serve as an important component of a cancer treatment strategy or cancer treatment adjuvant, such as radiotherapy [63, 64]. The role of RT in promoting apoptosis has been reported in a number of studies [65]. The JNK protein belongs to the stress-activated protein kinase family, which was originally discovered primarily through its ability to phosphorylate c-Jun, which is recognized as a key regulator of cell proliferation, cell survival, cell death, DNA repair, and metabolism [66].

c-Jun is the prototype substrate of JNK and an important part of JNK pathway, which can indirectly reflect the activity of JNK. c-Jun plays an important role in the activation of activator protein 1 (AP-1) complex, and plays an important role in the transformation process of cell proliferation and differentiation [67]. JNK binds and phosphorylates c-Jun and increases its transcriptional activity, thereby activating an important component of the AP-1 transcription complex, which is an important regulator of gene expression, the c-Jun protein is a widely studied member of the AP-1 family, and its functions include cell proliferation, apoptosis, growth, tumor formation, trauma, and stress, especially the stress caused by ionizing radiation [67, 68]. Bcl-2, as a downstream target gene of the JNK/c-Jun pathway, is involved in mediating and regulating cell apoptosis [69, 70].

The Bcl-2 gene belongs to the Bcl-2 family of genes that function to inhibit cell apoptosis and prolong the life of cells. The Bcl-2 gene is associated with tumor occurrence, development, and prognosis [71, 72]. It is known that various human hematological malignancies and solid tumors are highly expressed with the Bcl-2 proto-oncogene. The Bcl-2 protein acts as an oncogene by preventing tumor cells from undergoing apoptosis in response to radiation, chemotherapy, or hormonal therapy [73]. However, there are reports that although the upregulation of Bcl-2 is associated with the inhibition of cell apoptosis, it is associated with a good prognosis in the analysis of clinical data [74]. Breast cancer patients with a positive Bcl-2 protein expression are considered to have a better prognosis [75], and Bcl-2 is significantly associated with several favorable prognostic factors such as tumor size, ER and progesterone receptor positivity [73, 76]. The association between Bcl-2 expression and good survival may be explained by many factors, including the close relationship between Bcl-2 and hormone receptors, as well as by the interaction between other genes, such as the p53 gene. However, other studies suggest that the survival advantage associated with Bcl-2 expression may disappear after 10 years of follow-up, and long-term follow-up may be required to fully assess the prognostic significance of Bcl-2 protein expression [75, 77, 78]. Despite the above controversies, the current mainstream view is that Bcl-2 is an oncogene and plays an important role in the resistance of various forms of cancer treatment, such as resistance to chemotherapy and RT resistance [79, 80]. Our study found that downregulation of PDIA4 enhanced the apoptotic effect of radiotherapy in TNBC, and this was associated with the inhibition of Bcl-2 expression and increased of Bax.

PDIA4 belongs to the protein disulfide isomerase family, which is a key step in protein folding in the endoplasmic reticulum, and is involved in the formation of inter-protein disulfide bonds, which plays a significant role in promoting cancer [34]. Previous studies have shown that PDIA4 can mediate drug resistance by affecting apoptosis and DNA repair mechanism [81, 82]. DNA damage is one of the important effects of IR on cancer cells, and the inability of cancer cells to repair DNA damage is a characteristic of the effectiveness of RT. Therefore, the knockdown of PDIA4 enhances TNBC sensitivity of RT, which may be due to the regulation of JNK, Bcl-2, Bax expression damage, which leads to the increase of apoptosis index DNA.

During this study, we observed increased expression of PDIA4 in TNBC tissues and cell lines. In vitro experiments involving PDIA4 downregulation resulted in a significant reduction of proliferation and increased the apoptosis of tumor cells after RT as well as increasing its sensitivity of RT and vice versa. In addition, experiments in vivo revealed that the downregulation of PDIA4 induced apoptosis and inhibited the growth of TNBC as well as increasing its sensitivity of RT.

A variety of solid tumors have been reported to overexpress TAX1BP1, including breast cancer [83], lung cancer [84], and liver cancer [85]. Through the effects of the signal transduction pathway of NF-kB, JNK and other signaling pathways, TAX1BP1 plays a wide range of physiological and pathological roles, including cell apoptosis, embryonic development and immune response [43, 45], particularly in chemoresistance and RT resistance [86, 87]. According to recent studies, the JNK signaling pathway is crucial for regulating the sensitivity of RT in tumor cells [88, 89]. Further investigation revealed that PDIA4 could bind to TAX1BP1, where it would subsequently be proteasomal degraded. A series of experiments demonstrated that the biological function of PDIA4 in TNBC RT resistance depends on TAX1BP1-mediated inhibition of JNK.

## Conclusions

**Fig. 8:**
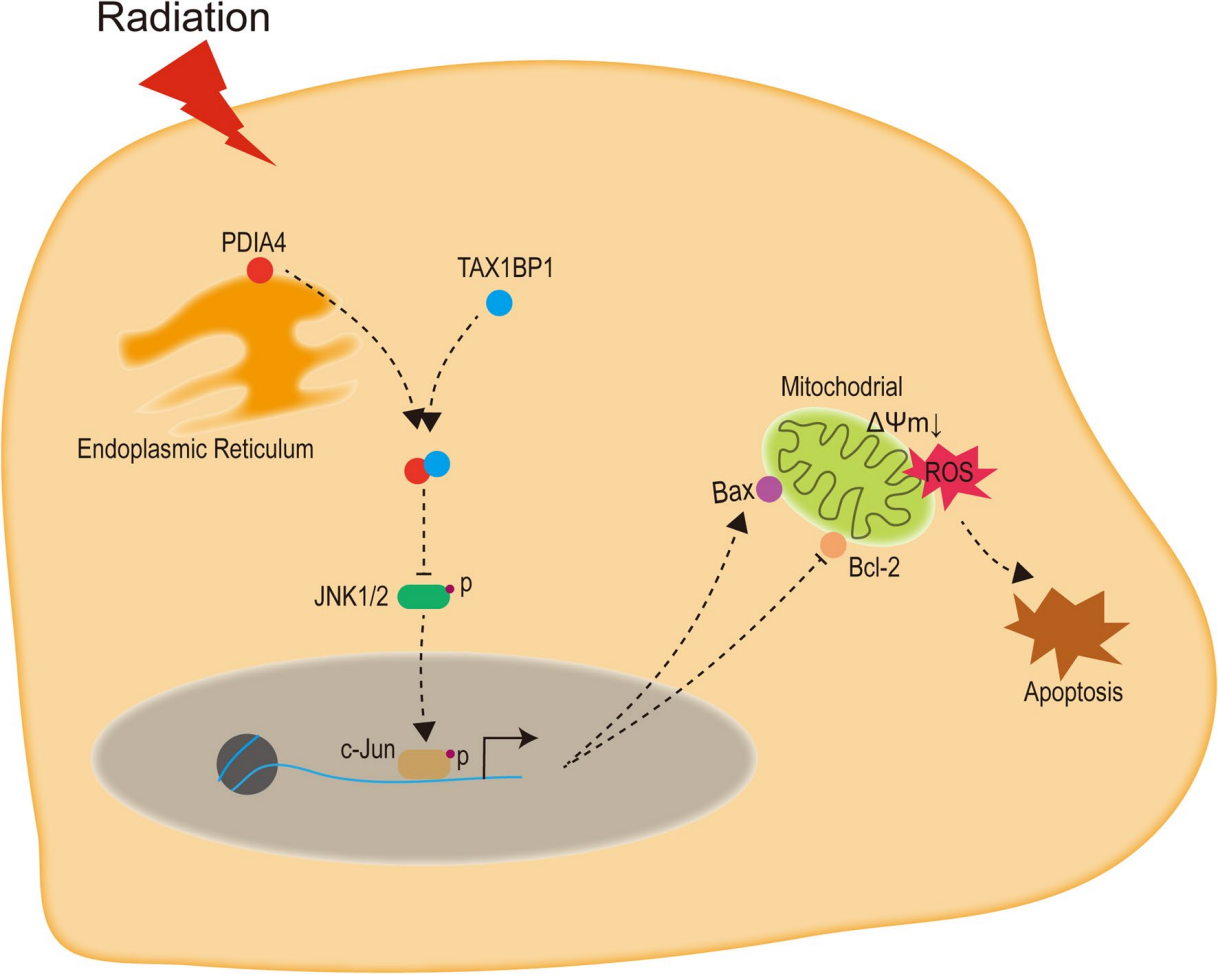
A schematic graph showing the possible mechanism of PDIA4 in TNBC tumorigenesis and RT resistance

In conclusion, we have identified a new target of TNBC acquired RT resistance. Loss of PDIA4 overcomes RT resistance both in vitro and in vivo, which may significantly improve the prognosis of TNBC patients, especially those who are resistant to RT. Results from the study provide evidence of the clinical relevance and role of PDIA4 in RT resistance in TNBC. Experimental studies in vitro and in vivo, we have demonstrated that PDIA4 promotes the malignancy of TNBC by inhibiting JNK through TAX1BP1 (Fig. 8), which may provide a therapeutic target for the treatment of this disease.

### Supplementary Information

**Additional file 1. Table S1. **Primer sequences for shRNA.

**Additional file 2. Table S2. **The primer sequences for qPCR.

**Additional file 3. Fig. S1**: PDIA4 mRNA expression levels in 33 different tumor types from TCGA database via GEPIA2 portal.

## Data Availability

The data used and/or analyzed during the study are available from the corresponding author on reasonable request.
