# Supplementary material for: Protein disulfide isomerase family member 4 promotes triple-negative breast cancer tumorigenesis and radiotherapy resistance through JNK pathway
Source: Breast Cancer Res. 2024 Jan 2;26:1. doi: 10.1186/s13058-023-01758-6 (PMC10759449; doi:10.1186/s13058-023-01758-6)
Supplement: Supplementary file 1 — Additional file 1. Table S1. Primer sequences for shRNA. [file 13058_2023_1758_MOESM1_ESM.docx]

**Supplementary Table S1. Primer sequences for shRNA**

| shRNAs | Primer Sequence |
| --- | --- |
| shPDIA4-1  (shRNA1) | F: gatccGCAAGCGTTCTCCTCCAATTCtcaagagGAATTGGAGGAGAACGCTTGCtttttt |
|  | R: aattaaaaaaGCAAGCGTTCTCCTCCAATTCctcttgaGAATTGGAGGAGAACGCTTGCg |
| shPDIA4-2  (shRNA2) | F: gatccGCCAAGAGGTTTGATGTCTCTtcaagagAGAGACATCAAACCTCTTGGCtttttt |
|  | R: aattaaaaaaGCCAAGAGGTTTGATGTCTCTctcttgaAGAGACATCAAACCTCTTGGCg |
| shPDIA4-3  (shRNA3) | F: gatccGCAAAGTTCTTGAAAGTCTCCtcaagagGGAGACTTTCAAGAACTTTGCtttttt |
|  | R: aattaaaaaaGCAAAGTTCTTGAAAGTCTCCctcttgaGGAGACTTTCAAGAACTTTGCg |
