# Supplementary material for: Protein disulfide isomerase family member 4 promotes triple-negative breast cancer tumorigenesis and radiotherapy resistance through JNK pathway
Source: Breast Cancer Res. 2024 Jan 2;26:1. doi: 10.1186/s13058-023-01758-6 (PMC10759449; doi:10.1186/s13058-023-01758-6)
Supplement: Supplementary file 2 — Additional file 2. Table S2. The primer sequences for qPCR. [file 13058_2023_1758_MOESM2_ESM.docx]

| Primer | | Sequence 5’-3’ |
| --- | --- | --- |
| PDIA4 | forward | AAGCGTTCTCCTCCAATT |
|  | reverse | GGACTGCTCGATCATGTAA |
| β-actin | forward | CAGAAGGAGATTACTGCTCTGGCT |
|  | reverse | TACTCCTGCTTGCTGATCCACATC |

**Supplementary Table S2. The primer sequences for qPCR**
