# Supplementary figures and images for: Protein disulfide isomerase family member 4 promotes triple-negative breast cancer tumorigenesis and radiotherapy resistance through JNK pathway
Source: Breast Cancer Res. 2024 Jan 2;26:1. doi: 10.1186/s13058-023-01758-6 (PMC10759449; doi:10.1186/s13058-023-01758-6)

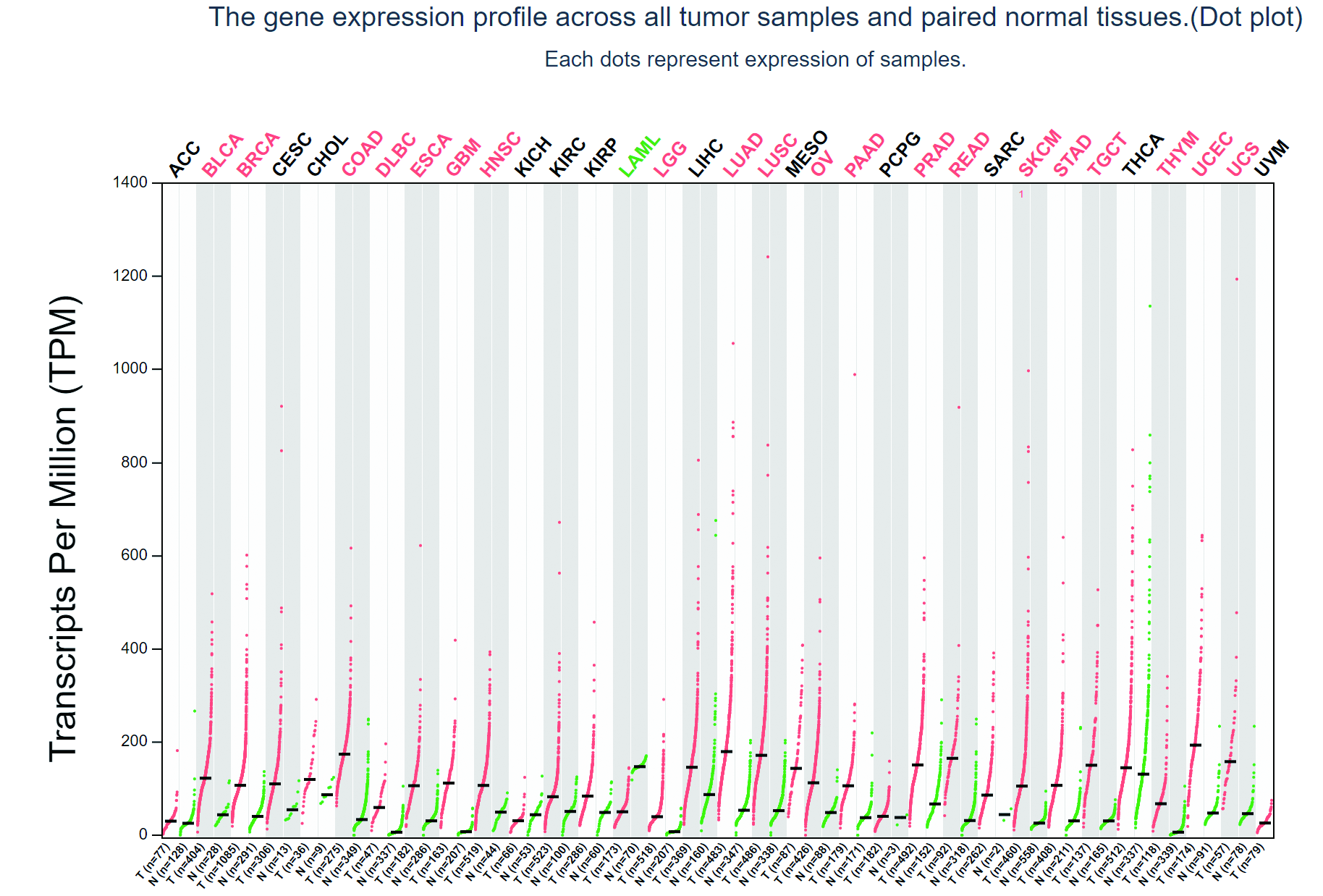

Supplement: Supplementary file 3 — Additional file 3. Fig. S1: PDIA4 mRNA expression levels in 33 different tumor types from TCGA database via GEPIA2 portal. [file 13058_2023_1758_MOESM3_ESM.tif]
